# Does Preference for Abstract Patterns Relate to Information Processing and Perceived Duration?

**DOI:** 10.1177/2041669515604436

**Published:** 2015-09-30

**Authors:** Letizia Palumbo, Ruth Ogden, Alexis D J Makin, Marco Bertamini

**Affiliations:** Department of Psychological Sciences, University of Liverpool, UK Department of Psychology, Liverpool Hope University, UK; School of Natural Sciences and Psychology, Liverpool John Moores University, UK; Department of Psychological Sciences, University of Liverpool, UK

**Keywords:** Clicks, arousal, fluency, timing, symmetry, preference

## Abstract

Repetitive prestimulation, in the form of click trains, is known to alter a wide range of cognitive and perceptual judgments. To date, no research has explored whether click trains also influence subjective preferences. This is plausible because preference is related to perceptual fluency and clicks may increase fluency, or, because preference is related to arousal and clicks may increase arousal. In Experiment 1, participants heard a click train, white noise, or silence through headphones and then saw an abstract symmetrical pattern on the screen for 0.5, 1, or 1.5 s. They rated the pattern on a 7-point scale. Click trains had no effect on preference ratings, although patterns that lasted longer were preferred. In Experiment 2, we again presented a click train, silence, or white noise but included both symmetrical and random patterns. Participants made both a duration and a preference judgment on every trial. Auditory click trains increased perceived duration, and symmetrical patterns were perceived as lasting longer than random patterns. Again there was no effect of auditory click trains on preference, and again patterns that were presented for longer were preferred. We conclude that click trains alter perceptual and cognitive processes, but not preferences. This helps clarify the nature of the click train effect and shows which predictions implicit in the existing literature are supported.

## Introduction

Past investigations have shown that auditory click trains (repetitive auditory signals at about 5 Hz) alter subsequent processing (Jones, 2014). Tones or visual presentations are judged as longer in duration when preceded by click trains than silence (Droit-Volet, 2010; Droit-Volet & Wearden, 2002; Penton-Voak, Edwards, Percival, & Wearden, 1996; Treisman, Faulkner, Naish, & Brogan, 1990; Wearden, Philpott, & Win, 1999). More recently, it has been shown that magnitude estimations for various dynamic displays are also increased by the prior presentation of click trains (Droit-Volet, 2010), and that click trains increase the subjective velocity of moving stimuli (Makin et al., 2012; Makin, Lawson, Bertamini, & Pickering, 2014). Click trains have also been shown to enhance wider cognitive processing: Jones, Allely, and Wearden (2011) demonstrated that click trains can increase the amount of mental arithmetic that can be completed within a given duration, and the number of items recalled from a rapid digit presentation.

Cognitive timing mechanisms have been modeled and tested extensively, and the effect of click trains on subjective duration has been considered within these frameworks. According to *Scalar Expectancy Theory*, specific clock, memory, and decision modules produce timed behavior. The clock has subcomponents: a pacemaker that emits “ticks” at regular intervals and an accumulator, which stores the number of ticks. When time becomes task relevant, a switch closes and ticks begin accumulating (Buhusi & Meck, 2005; Gibbon, Church, & Meck, 1984; Wearden, 1991). Let’s briefly consider an example from a typical cognitive timing task. On each trial, the participant is presented with a test duration, and then they estimate whether it was longer or shorter than a well-learned standard duration that they have memorized. The pacemaker emits ticks, say at 10 Hz. When the onset of the test stimulus is detected, the switch closes, and ticks start passing from the pacemaker into the accumulator. When the offset of the test stimulus is detected, the switch opens, and the accumulation process is terminated. The final number of ticks in the accumulator is proportional to an elapsed duration. This can then be compared with the number of ticks associated with the standard duration in memory, and the decision is made.

The rate at which the pacemaker emits ticks is thought to be arousal sensitive, with greater arousal producing faster output and longer perceptions of duration (Meck, 1983). Indeed, arousing stimuli, such as fear-inducing images (Droit-Volet & Meck, 2007), pain (Ogden, Moore, Redfern, & McGlone, 2015), drugs (Meck, 1983), and increases in body temperature (Wearden & Penton-Voak, 1995), elongate subjective duration. One possibility, therefore, is that click trains produce arousal, leading to an increase in pacemaker speed, and a longer perceived duration for the stimulus that follows them (Treisman et al., 1990).

An alternative suggestion is that click trains speed up the rate at which information is processed (Jones et al., 2011). It is possible that duration estimates are based on the amount of information processed within an interval (Kanai, Paffen, Hogendoorn, & Verstraten, 2006). Therefore, the effect of clicks on subjective duration could be mediated by effect of clicks on information processing rate. Click trains may produce a driven alpha rhythm across many brain areas, and this could be responsible for subsequent alterations in information processing rate (see Jones et al., 2011 for discussion).

While the effect of click trains on subjective duration and other perceptual judgments is well documented, no research to date has explored whether clicks also influence subjective preferences. The idea that click trains could have an effect on preference is plausible. One possibility is that clicks influence preference directly (in the same sense that they putatively increase pacemaker speed directly).

However, the effect of clicks on preference could also be mediated indirectly. The influential *fluency hypothesis* states that people are sensitive to the efficiency and speed of their own perceptual and cognitive processes. The feeling of high-processing fluency has positive hedonic tone, and this can be attributed to the goodness of the stimuli under some circumstances (Reber, 2012). Clicks increase the amount of information processed within a duration (Jones et al., 2011), and this enhanced information processing could be akin to higher fluency. The greater fluency could then increase liking, as has been repeatedly observed. Another pathway is through arousal. It has long been proposed that arousal is related to preference (Berlyne, 1971). If clicks increase arousal (as proposed by Treisman et al., 1990), then they could also alter preferences. In sum, there are at least three nonexclusive mechanisms by which clicks could alter preferences (directly, through fluency, or arousal), but the basic effect has never been demonstrated.

## Experiment 1

In Experiment 1, we presented different auditory stimuli (click trains, silence, or white noise) followed by checkerboards with a symmetrical configuration. We chose symmetrical stimuli because they are appealing (e.g., Makin, Pecchinenda, & Bertamini, 2012), and this could make participants engage with the preference task. We asked participants to rate preference on a 1- to 7-point scale (1 = *unattractive* to 7 = *attractive*).

**Figure 1.**
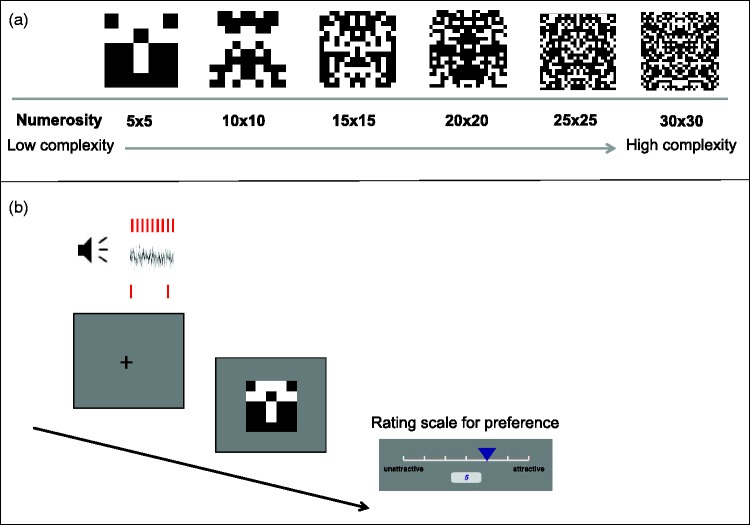
Experiment 1 method. (a) Illustration of the six types of matrices from low complexity (5 × 5) to high complexity (30 × 30). (b) Each trial started with a fixation cross, while an auditory stimulation (clicks or white noise or silence) was presented for 4 s. A symmetrical pattern was presented for one of three possible durations in seconds (0.5, 1.0, 1.5). Participants rated preference for the pattern on a 7-point scale: 1 = *unattractive*, 7 = *attractive*.

We manipulated the complexity of the patterns by varying the size and number of elements, and the duration of the patterns by varying exposure (0.5, 1, or 1.5 s; Figure 1). This had the advantage of adding variability to the stimuli, but also it allowed us to explore possible interactions between sound type and other features that could alter preference. The effects of complexity, order, and arousal on aesthetics preference have been well studied (although many questions remain, for a recent review see van Tonder & Spehar, 2013), so it is possible that the effect of clicks on preference could be moderated by complexity or duration of the patterns in a systematic way. Experiment 1 allowed us to explore these possibilities.

### Method

#### Participants

Forty-two participants took part in the experiment (age range 17–55; 39 right handed; 30 females), and all had normal or corrected to normal vision. They provided a written consent for taking part and received a reimbursement for their time or course credits. The experiment was approved by the Ethics Committee of the University of Liverpool and was conducted in accordance with the Declaration of Helsinki (2008).

#### Stimuli and apparatus

Participants sat in a quiet room at approximately 57 cm of distance from the screen. The sounds were played through Labtec LCS-1021 loud speakers. The visual stimuli were presented on a 1280 × 1024 DELL M993s 19″ cathode ray tube monitor at 60 Hz. The auditory stimuli consisted of three different sounds played for 4 s prior to the visual pattern: click trains (5 Hz, 16 bits), white noise (16 bits), and silence, the latter was delimitated by two separate clicks (start and end). We included the white noise condition as a control because click trains may have a specific effect or simply act like a strong warning cue that the trial is about to start (Penton-Voak et al., 1996). Previous work has repeatedly demonstrated that prestimulus click trains of this type alter subjective duration and other perceptual judgments, even when the clicks are not attended (e.g., Droit-Volet, 2010; Jones et al., 2011; Penton-Voak et al, 1996; Wearden et al., 1999).

The stimuli were created in Psychopy software (Peirce, 2007). The visual stimuli consisted of a matrix (320 × 320 px; ∼10 × 10degrees of visual angle) with a number of black or white squares ranging from 25 (5 × 5) to 900 (30 × 30). Position of checks was assigned as to form reflectional symmetry around a vertical axis. The symmetric configuration of the items changed from pattern to pattern, whereas the black and white ratio was the same as to keep luminance and surface area constant (Figure 1). This means that the size of the items was inversely related to numerosity. There were six types of matrices, the difference in numerosity served as a measure of complexity (see Figure 1(a)).

#### Experimental design and procedure

We employed a 3 × 3 × 6 within-subjects design with as factors sound (clicks, white noise, silence), duration (0.5, 1.0, 1.5 s), and visual complexity (5 × 5, 10 × 10, 15 × 15, 20 × 20, 25 × 25, 30 × 30 items). The dependent variable was the attractiveness judgment.

The experiment started with the instructions followed by the practice (six trials presented in random order across participants). As in previous studies, participants were instructed to attend to all experimental events but were not instructed to specifically process the clicks themselves (e.g., see Droit-Volet & Wearden, 2002; Jones et al., 2011; Makin, et al., 2012; Makin et al., 2014). Each trial started with a black fixation cross at the center of a gray background, while one of the three sounds was played for 4 s. After the sound, there was a randomized 0.5 to 1 s delay before the pattern appeared. The symmetric pattern was displayed for one of the three possible durations (0.5, 1.0, 1.5 s). After this, the 7-point scale, ranging from *unattractive* (1) to *attractive* (7), appeared on the bottom of the screen. Participants provided response by a mouse click on the scale (see Figure 1(b)).

The practice was followed by 6 blocks of 18 experimental trials, each one of which were identical to the practice with the exception that novel patterns were presented. Participants were encouraged to take a break at the end of each block. The experiment lasted approximately 30 min.

#### Analysis

Mean preference estimates were obtained in each condition for each participant. These data points were then analyzed with a three factor repeated measures analysis of variance (ANOVA). The Greenhouse-Geisser correction factor was applied, when the assumption of sphericity was violated. We report partial η^2^ values following significant effects.

### Results

**Figure 2.**
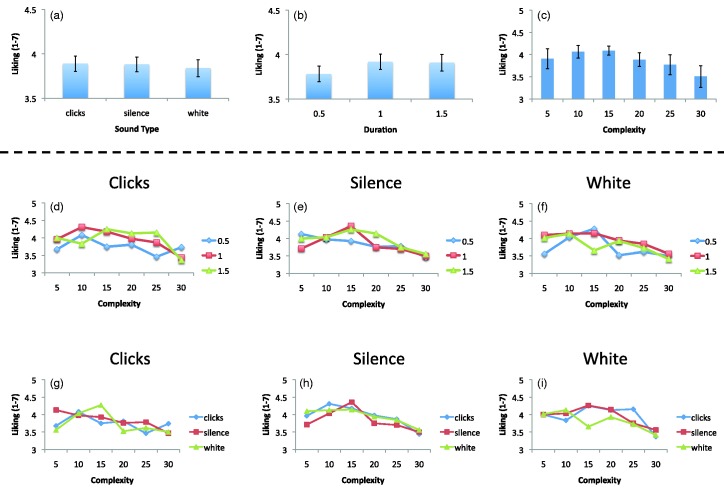
Preferences in Experiment 1. Panels a to c show main effects. Panels d to i show various interactions. Although there were some complicated two-factor interactions, there was no systematic subset of conditions where sound influenced preference.

The results are illustrated in Figure 2. Sound type had no effect on preference judgments, *F*(2, 82) = .645, *p* = .527, Figure 2(a). There was a significant main effect of duration, *F*(1.65, 67.76) = 4.33, *p* = .023, $\eta_{\text{p}}^{2}$ = .096, Figure 1(b), because patterns were liked more when they stayed on screen for a longer time (0.5 vs. 1.0 s: *t*(41) = 3.300, *p* = .002; 0.5 vs. 1.5 s: *t*(41) = 2.063, *p* = .045, Figure 2(b)). There was no main effect of complexity, *F*(1.23, 5.43) = 1.325, *p* = .255, Figure 2(c).

There was no Sound × Complexity interaction, no Sound × Duration interaction no Complexity × Duration interaction: (*p* > .059 in all cases). However, the three-way interaction between Sound × Visual Complexity × Duration was significant, *F*(11.33, 464.56) = 2.865, *p* = .001, $\eta_{\text{p}}^{2}$ = .065. As shown in Figure 2(h), there were a range of different two-factor interactions, some of which were significant. Rather than list all these, we consider the broader research question. Is there a particular subset of conditions where clicks have a systematic effect on preference? The answer is no. There is no clear case where preference ratings in the clicks condition diverge from the other two sound conditions.

### Discussion

Auditory click trains alter several subsequent perceptual magnitude judgments (Droit-Volet, 2010; Jones, 2014). We tested whether click trains would also alter preference for abstract symmetrical patterns. This potential effect on preference could be mediated directly, through increases in perceptual fluency, or through increased arousal. However, contrary to our predictions, we found that auditory click trains had no influence on preference for symmetrical patterns. There was a three-way interaction between sound type, duration, and complexity. However, there was clearly no subset of conditions in which clicks had a unique effect on preference. This suggests that the effect of click trains is specific to perceptual and cognitive processes, and that clicks do not alter evaluations, either directly or indirectly.

In Experiment 1, we only obtained preference judgments and just assumed that click trains were having their usual effect on perceptual and cognitive processing. In Experiment 2, we verified that click trains were indeed having the expected effect on duration by obtaining both duration and preference judgments on every trial. This rules out a potential explanation for our null results in Experiment 1; it could be that click trains atypically failed to alter perceptual processes; hence, changes in preference which would have been mediated by the altered perceptual processing failed to occur. Another possible explanation for the null effect of clicks on preference in Experiment 1 was that we only presented symmetrical stimuli, which are generally evaluated favorably. This could have masked the effect of click trains.

The only significant main effect in Experiment 1 was that of duration. Participants liked less the symmetrical stimuli presented for 0.5 s compared with those presented for 1 or 1.5 s (Figure 2(b)). In Experiment 2, we reexamined this in more detail. Experiment 2 compared symmetrical and random patterns. It is well known that symmetry is preferred to random, and this has been shown with explicit ratings measures like those used in Experiment 1 (Eisenman, 1967; Eysenk, 1941; Jacobsen, 2002) and with implicit measures of preference (Bertamini, Makin, & Pecchinenda, 2013; Makin et al., 2012). There are many, nonexclusive reasons why people like symmetry, including sexual selection (Grammer, Fink, Møller, & Thornhill, 2003), visual relevance (Machilsen, Pauwels, & Wagemans, 2009), or high-perceptual fluency (Makin et al., 2012). Why, then, did longer presentation durations increased preference for symmetry in Experiment 1? One explanation is that there was more time for positive evaluations and associations to form. Experiment 2 tested whether this effect is specific to symmetry. It could be that longer durations would enhance the difference between symmetry and random, so the preferred symmetry looks better, and the disliked random patterns look worse. Alternatively, longer presentation durations could lead to more positive evaluations in general, both for symmetry and random.

## Experiment 2

In Experiment 2, the auditory stimulation (click trains, silence, or white noise) was followed by the presentation of symmetric or random patterns, which stayed on screen for one of five different durations (0.5, 0.75, 1.0, 1.25, 1.5 s). We asked participants to perform two tasks on every trial. They both judged the appeal of the pattern and its duration. We expected that overall, symmetrical patterns would be preferred over the random ones. On the basis of the results of Experiment 1, we also predicted that preference for symmetrical patterns would increase with longer presentation durations. However, we did not know whether this would generalize to random patterns.

**Figure 3.**
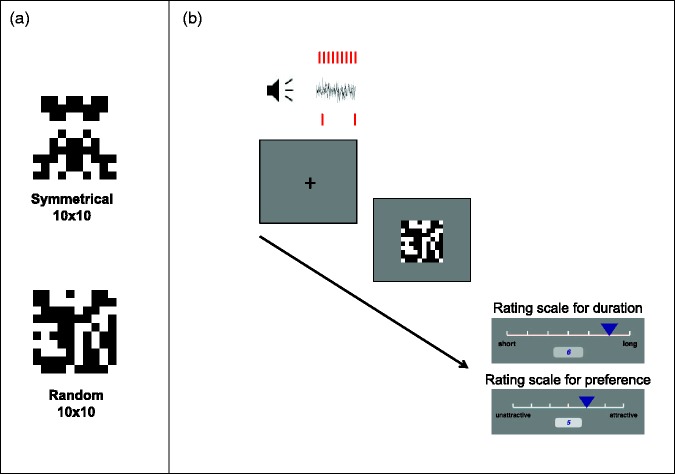
Experiment 2 method. (a) Illustration of type of stimuli (symmetrical vs. random). (b) Each trial started with a fixation cross, while an auditory stimulation (clicks or white noise or silence) was presented for 4 s. A symmetrical or random pattern was presented for one of five possible durations in seconds (0.5, 0.75, 1.0, 1.25, 1.50). On every trial, participants rated preference for the pattern and estimated pattern durations.

On the basis of the results of Experiment 1, we did not expect clicks to have any effect on preference in the symmetrical condition. There may, however, be an effect of click trains in the random condition. On the basis of extensive previous literature, we did expect click trains to increase perceived duration. This would suggest that the null effect in Experiment 1 could not be attributed to the unusual absence of an influence of click trains on perceptual processes. Complexity was not manipulated in Experiment 2. Every pattern was a 10 × 10 black and white matrix (Figure 3). Our previous work shows that this kind of image complexity has no effect on subjective duration (Palumbo, Ogden, Makin, & Bertamini, 2014).

### Method

#### Participants

Twenty-four participants took part in the experiment (age range 19–25; 2 left handed; 19 females), and all had normal or corrected to normal vision. They provided a written consent for taking part and received a reimbursement for their time or course credits. The experiment was approved by the Ethics Committee of the University of Liverpool and was conducted in accordance with the Declaration of Helsinki (2008).

#### Stimuli and apparatus

The auditory stimuli were the same as in Experiment 1 (5 Hz clicks, white noise and silence presented for 4 s). Stimuli consisted of a matrix with 10 × 10 squares (320 × 320 px, each square 32 × 32 px). Position of the squares formed a vertical reflection or a random configuration (Figure 3(a)).

#### Experimental design and procedure

We employed a 3 × 2 × 5 within-subjects design with as factors sound (clicks vs. white noise vs. silence), regularity (symmetry vs. random) and duration (0.5, 0.75, 1.0, 1.25, 1.5 s).

The experiment started with the instructions followed by the practice (six trials). As in Experiment 1, each trial started with a black fixation cross, showed at the center of a gray background, while one of the three sounds was played for 4 s. After the sound, there was a randomized 0.5 to 1 s delay before the pattern appeared. Following this, the pattern was displayed for one of the five possible durations. Participants estimated pattern duration by selecting with a mouse click one on a 7-point scale (1 = *short* to 7 = *long*). As in Experiment 1, they performed the preference task selecting one option on the 7-point scale (1 = *unattractive* to 7 = *attractive*, Figure 3(b)). The order of the duration and preference ratings was counterbalanced across participants. Four blocks of 30 experimental trials, presented in a randomized order, followed the practice. The experimental trials were identical to the practice ones with the exception that novel patterns were shown. The experiment lasted approximately 45 min.

### Results

#### Duration estimates

**Figure 4.**
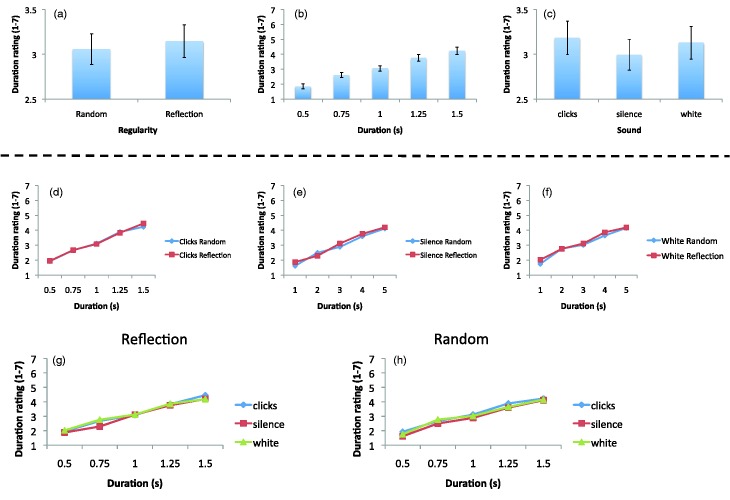
Duration estimations from Experiment 2. Panels a to c show main effects. Note that there were significant differences in all cases, even though error bars are relatively large. Panels d to h indicate that there were no interaction effects.

The effects for subjective duration are illustrated in Figure 4. We found that regularity altered subjective duration. Symmetry was judged to have lasted longer than random patterns, *F*(1, 23) = 5.332, *p* = .028, $\eta_{\text{p}}^{2}$ = .194, Figure 4(a). Unsurprisingly, there was significant main effect of duration, *F*(1.24, 28.52) = 79.28, *p* = .000, $\eta_{\text{p}}^{2}$ = .775, because our participants could estimate duration reasonably accurately (Figure 4(b)). There was also a main effect of sound, *F*(2, 46) = 3.81, *p* = .029, $\eta_{\text{p}}^{2}$ = .142, Figure 4(c). As expected, patterns that followed the train of clicks were judged as lasting longer, then the patterns that followed the silence, *t*(23) = 3.31, *p* = .003. Although there was no difference between silence and white noise, *t*(23) = 1.699, *p* = .103, there was also no difference between clicks and white noise, *t*(23) = 0.732, *p* = .472. Therefore, clicks did not have a unique effect on subjective duration, as predicted by previous work. There were no interaction effects (*p* > .167 in all cases). This is important because it indicates that there were no differences in the slope relating duration to estimated duration in any conditions.

#### Preferences

**Figure 5.**
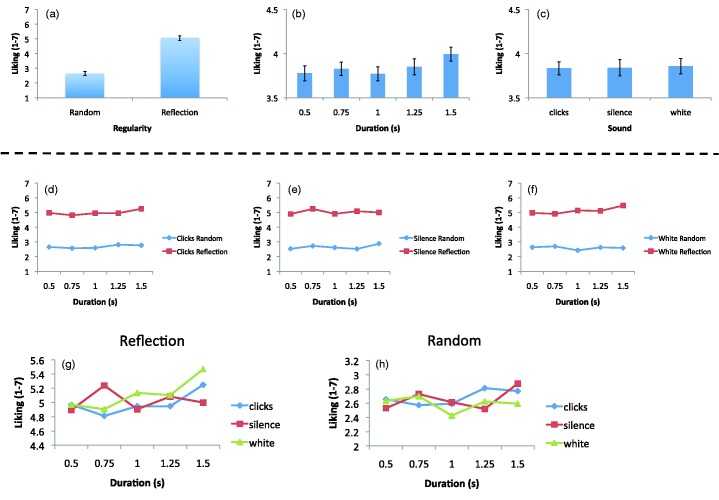
Preferences in Experiment 2. Panels a to c show main effects. Panels d to h show various interactions. As with Experiment 1, although there were some complicated two-factor interactions, there was no systematic subset of conditions where sound influenced preference.

Preference results are shown in Figure 5. There was a main effect of regularity, *F*(1, 23) = 80.79, *p* = .000, $\eta_{\text{p}}^{2}$ = .778, Figure 5(a), because symmetric patterns were liked more than random patterns. The main effect of duration was also significant, *F*(4, 92) = 6.613, *p* = .000, $\eta_{\text{p}}^{2}$ = .223 because preference increased with duration Figure 5(b). As in Experiment 1, there was no main effect of sound on preference, *F*(1.31, 30.18) = 0.068, *p* = .860, Figure 5(c). The preference for longer presentation durations was found in both symmetry and random conditions: There was no Regularity × Duration interaction, *F*(4, 92) = 0.955, *p* = .420. This suggests that longer presentation durations do not amplify preexisting valence but make both positive and negative stimuli seem slightly more positive.

Finally, the three-way interaction between sound, regularity, and duration was also significant, *F*(8, 184) = 3.138, *p* = .002, $\eta_{\text{p}}^{2}$ = .120. The overall effect of preference for symmetry over random was the dominant effect in all three sound conditions, and at all levels of duration, with some relatively minor fluctuations (Figure 5(d) to (f)). However, there were disparate interactions between sound and duration in the symmetry and random conditions, which is shown in Figure 5(g) and (h). As with Experiment 1, there was no clear subset of conditions where clicks altered preference systematically. Instead, preference ratings from the clicks condition generally lay among those of the silence and white noise conditions. Figure 5(g) and (h) shows that the increase in preference with duration was present in most conditions but was not found in the silence-symmetry condition or in the white noise-random condition.

### Discussion

Experiment 2 showed that prior presentation of click trains increased subjective duration, at least compared with silence. This replicates all previous work in this area (Droit-Volet, 2010; Jones et al., 2011; Penton-Voak et al., 1996; Treisman et al., 1990). As with Experiment 1, there was no effect of auditory stimulation on preference. Experiment 2 thus shows dissociation between the effect of clicks on time perception and the effect of clicks on preference, even though there are theoretical reasons to expect a similar pattern of results in both cases.

In Experiment 1, we found that longer presentations led to higher preference ratings: Experiment 2 replicated this effect and showed that it was common to both the preferred symmetrical patterns and the disliked random patterns. Therefore, long durations did not exaggerate existing preferences, but rather increased liking in both. Preference for symmetry over random far outweighed the much smaller effect of duration. This is unsurprising: Symmetry is routinely found to be a very strong predictor of explicit and implicit preference for abstract patterns (Makin et al., 2012).

There were two unexpected findings involving subjective duration. In most experiments, the absolute size of the click train influence grows with stimulus duration. In other words, the slope of the relationship between actual duration and estimated duration is usually steeper in the click train condition than the silence condition. This is often explained by the idea that clicks increase the pacemaker speed in a pacemaker-accumulator clock (Penton-Voak et al., 1996). However, we did not find these slope effects on duration estimates in Experiment 2. Although slope effects are typically observed following click trains, and a recent study by Jones and Ogden (2015) also only observed an additive effect of clicks, indicating that slope effects may not be ubiquitous. Second, there was no significant difference between click trains and white noise. In previous work, the effect of clicks has been shown to be significantly greater than white noise, while white noise is similar to silence (Makin et al., 2012; Penton-Voak et al., 1996). This casts some doubt on the reliability of previous work on click trains and temporal judgments. It could be that the dual task of considering duration and preference altered the usual pattern of duration estimates (while leaving some effects of clicks intact).

Another novel result in Experiment 2 was that symmetrical patterns were judged to have lasted longer than random ones, as well as being liked more. These two effects may be linked, as the time perception literature has reported many variations of subjective time with emotional stimuli (Angrilli, Cherubini, Pavese, & Manfredini, 1997; Droit-Volet & Gil, 2009; Ogden, 2013; Ogden et al., 2015). It is possible that the positive valence of symmetry increases subjective duration of symmetry or that the negative valence of random decreases the subjective duration of random, further investigation is warranted.

#### Confirming the null hypothesis?

Perhaps the predicted effect of clicks on preference would have been significant if the experiment had greater power? It is notoriously problematic to base conclusions on nonsignificant effects. To overcome these traditional limitations, we reexamined the effect of clicks using a new Bayesian alternatives to null hypothesis testing (Masson, 2011). These procedures give two probability values, one for the null hypothesis and one for the alternative hypothesis (rather than simply a single probability for the observed data given the null hypothesis). In Experiment 1, the null hypothesis that there would not be effect of sound on preference has a probability estimate of 96%. Conventions are less well established than with convention statistics, but probabilities more than 90% are considered strong, >99% = very strong. For Experiment 2, the same procedure gives the null hypothesis of probability of 86% and the alternative hypothesis a probability of 14%. This approach to statistics supports our claim that clicks have no effect on preference. For comparison, we can analyze the known effect where clicks elevate subjective duration compared with silence. Given the observed data, the null hypothesis is given a probability of only 4.2%, which the alternative hypothesis is assigned 95.8%.

## General discussion

There are various intriguing relationships between auditory click trains, temporal judgments, subjective preference, and regularity. Some of these relations are implicit in existing literature, while others have already been tested. This work fills some gaps in the current empirical work.

First, evaluations of abstract stimuli are often shaped by the perceptual fluency. People like patterns that are more fluently processed. Specifically, perceptual fluency has positive hedonic tone, and this positive affect can be attributed to the stimuli under some conditions (Reber, 2012). Perceptual fluency has been operationalized as faster detection speed, and abstract patterns that are detected quicker are usually those that are preferred (Makin et al., 2012).

Prior presentation of auditory click trains may increase perceptual fluency. Clicks (5 Hz for 3–5 s) have been shown to increase the amount of information processing that can occur within a given duration and increase subjective duration. We hypothesized that such click trains should increase liking of abstract patterns. However, this was not found in Experiments 1 or 2. This is despite the fact that the clicks did increase the subjective duration of the same stimuli in Experiment 2. Therefore, although the hypothesis that clicks should increase preferences had prior plausibility, it was not confirmed. Perhaps the magnitude of the increase in perceptual fluency caused by click trains was not strong enough to alter preference, or perhaps, for some reason, perceptual fluency was not attributed to the merits of the stimuli in this case. We also reasoned that clicks might produce arousal, which could also have knock on effects on preference judgments (Treisman et al., 1990, Berlyne, 1971). Clearly this did not occur either.

We cannot exclude the possibility that some other type of repetitive stimuli could have altered preferences, even though our auditory click trains did not. Our clicks were 5 Hz and lasted 4 s, while participants did not explicitly have to process the clicks. These parameters were very similar to those used in numerous interval-timing tasks, where positive effects of clicks on subjective duration have been reported (e.g., Droit-Volet, 2010; Jones et al., 2011; Treisman et al., 1990, Penton-Voak et al., 1996). However, it is possible that clicks of different frequency, duration, or task relevance could alter preference. Third, it is known that cross-modal attentional division can influence elements of visual processing (e.g., binocular rivalry, Alais, van Boxtel, Parker, & van Ee, 2010). Therefore, we cannot exclude that the use of a repetitive visual (rather than auditory) prestimulus would have altered preferences in our experiment. Some of these possibilities will have to be tested in future work.

The most influential work on interval timing literature has concluded that white noise has no effect on subjective duration, and that the effects of clicks on subjective duration are multiplicative, growing with stimulus duration (e.g., Penton-Voak et al., 1996). Duration estimates in our Experiment 2 were not consistent with either claim. We found no slope effect and no significant difference between clicks and white noise. Do these apparent anomalies mean that the data set cannot be the basis for solid conclusions? We think not. First, the slope effect for clicks on duration has not always been replicated (Jones & Ogden, 2015). Second, the absence of a click effect on preference was replicated in Experiments 1 and 2, so this cannot be dismissed as an anomaly. Third, the ambiguities about white noise and subjective duration do alter any of our conclusions about clicks and preference. At worst, white noise might alter subjective duration, and there might be nothing special about clicks. That would indeed challenge some earlier conclusions about the unique effect of repetitive prestimulation on perceptual judgments. However, it would not challenge our conclusion that repetitive prestimulation has no effect on preference for abstract patterns.

Despite the null effect of clicks on preference, this work produced other novel results. Both experiments found that stimuli that were presented longer were preferred. This was true for symmetrical patterns in Experiment 1 and for both symmetrical and random patterns in Experiment 2. The effect of duration on preference was not an amplification effect, where existing valence is exaggerated (Albrecht & Carbon, 2014). Instead, it seems that longer presentations are preferred uniformly. We hypothesize that having longer to process the stimuli is in itself rewarding, independently of stimulus valence.

Symmetrical patterns were judged to have lasted longer than random patterns. We can ask which module of this internal clock is responsible for this? As stated above, anything that alters pacemaker speed should produce different duration versus estimated duration slopes (Penton-Voak et al., 1996). However, this was not found, the slopes were similar in the symmetry and random conditions. This suggests symmetry and random presentations do not result in differential pacemaker speeds. Instead, it could be that the switch connected the pacemaker to the accumulator opened later in the random condition because attention was briefly focused away from time in this condition. Either would reduce the number of accumulated ticks in the random condition, resulting in the observed effect. Dual tasks have been shown to alter temporal judgments (Zakay, 1998): When attention is shifted to other tasks, subjective duration reduces, while when people attend to duration alone subjective duration increases (Macar, Grondin, & Casini, 1994). It could be that presentation of the random patterns shifted attention away from the timing task, so subjective duration was reduced, or equally, we could say, presentation of symmetrical patterns, which were processed with relative ease, allowed attention to remain on the timing task, so subjective duration was increased.

In summary, we have investigated several hypotheses implicit in the current literature on timing and symmetry perception. Despite prior plausibility, the presentation of auditory click trains does not increase preference for subsequently presented abstract patterns, even though the clicks do increase the perceived duration of the same patterns. We have, however, shown that preferred (symmetrical) patterns appear to have been presented for slightly longer than less preferred (random) patterns, and then longer presentation duration slightly increases preference. Subjective time and preference are intimately connected in the human brain, but not all plausible links are empirically demonstrable.
